# MTOR Inhibition Alters miRNA Profile in Cultured Bone Marrow Mesenchymal Stem Cells

**DOI:** 10.1093/geroni/igab046.2516

**Published:** 2021-12-17

**Authors:** Lisa Stukenborg, Manali Potnis, Christian Sell

**Affiliations:** 1 Drexel University College of Medicine, Philadelphia, Pennsylvania, United States; 2 Drexel University, Philadelphia, Pennsylvania, United States

## Abstract

Progressive decline in adult stem cell function is a feature of aged tissues and contributes to multiple age-related diseases. Of particular interest are bone marrow mesenchymal stem cells, also known as bone marrow stromal cells (BMSCs), which are multipotent, and have significant therapeutic potential for age-related disease. BMSCs isolated from older individuals show decreased differentiation and characteristics of cell senescence. We and others find that inhibition of the mTOR pathway prevents senescence and extends BMSC proliferation. We have examined miRNA profiles in rapamycin-treated BMSCs to identify miRNAs that may be needed to maintain “stemness” and facilitate differentiation in this population in undifferentiated cell populations. The analysis reveals a distinct miRNA profile induced by rapamycin associated with enhanced differentiation capacity.

